# Child suicides in Sweden, 2000–2018

**DOI:** 10.1007/s00431-021-04240-7

**Published:** 2021-09-02

**Authors:** Mensura Junuzovic, Kaja Maria Toporska Lind, Ulf Jakobsson

**Affiliations:** 1 Department of Community Medicine and Rehabilitation, Forensic Medicine, Umeå University, PO Box 7616, 907 12 Umeå, Sweden; 2 Department of Clinical Sciences in Malmö, Center for Primary Health Care Research, Clinical Research Centre, Lund University, Jan Waldenströms gata 35, 214 28 Malmö, Sweden; 3 Department of Pediatrics, Skåne University Hospital Lund, Entrégatan 7, SE-222 42, Lund, Sweden

**Keywords:** Suicide, Children, Suicide methods, Demographics

## Abstract

Although child mortality is decreasing in Sweden, an increase in suicide rates has been previously observed among children and adolescents collectively. To increase knowledge about trends, demographics, and means in child suicides, data including all child (< 18 years) suicides in Sweden in 2000 through 2018 were retrieved from the Swedish National Board of Forensic Medicine. In all, a total of 416 child suicides were found in a 19-year period, accounting for an annual suicide rate of 1.1/100,000 child population. The number of suicides increased with 2.2% by each successive year during the study period (*p* < 0.001). The mean age in both sexes was 16 years; boys accounted for 55% and girls for 45% of all study cases. The majority of the children who died by suicide (96%) were teenagers (13–17 years old) and suicides in children younger than 10 years were uncommon. Suicide methods were 59% hanging, 20% lying/jumping in front of a moving object, 8% jumping from a height, 7% firearm injury, 4% poisoning, and 2% other methods. Sex differences were significant (*p* < 0.001) only for firearms being preferably used by boys. The vast majority of firearms used were licensed long-barreled weapons.

*Conclusion*: The number of child suicides in Sweden is relatively low but increasing. Most of the children used a violent and highly lethal method. Prevention of premature mortality is an urgent concern with an emphasis on resolutely reducing the availability of suicide means.

**Table:** 

**What is Known:** *• Suicide is a significant cause of death globally among children, bringing tragic consequences for young individuals, their family, and the entire society.* *• Suicide rates and distribution of suicide methods in children differ between countries and settings, but studies of time trends are scarce.*	
**What is New:** *• Increasing number of minors’ suicides and the predominance of violent methods emphasize the importance of prevention strategies tailored for a child population.* *• Even in a setting of very restrictive firearm laws, firearm suicides in children must not be overlooked.*

**What is Known:**

*• Suicide is a significant cause of death globally among children, bringing tragic consequences for young individuals, their family, and the entire society.*

*• Suicide rates and distribution of suicide methods in children differ between countries and settings, but studies of time trends are scarce.*

**What is New:**

*• Increasing number of minors’ suicides and the predominance of violent methods emphasize the importance of prevention strategies tailored for a child population.*

*• Even in a setting of very restrictive firearm laws, firearm suicides in children must not be overlooked.*

## Introduction

Globally, suicides represent a significant health problem as the leading cause of death among 10–19-year-olds in low- and middle-income countries and the second leading cause of death in high-income countries in the European Region [1]. The rate of child suicides and its reporting varies between countries [2]. In the 5-year period (2014–2018), the suicide rate among 15–19-year-olds in Sweden (6.9 per 100,000 people aged 15–19 years) was higher than in Denmark (3.7) and Germany (4.7), but lower than in Finland (8.6) [3]. Among European countries, the highest rates for the same age group and period were reported in Estonia (13.3) and Iceland (17.0), while Greece had the lowest rate of 1.5 [3].

According to the WHO, depression and anxiety disorders are among the top five causes of overall disease burden in children and adolescents in the WHO European Region [1]. Furthermore, mental health issues in Swedish children and youths have increased in recent decades [4], as have suicide attempts and suicides among 15–24 year-olds [5]. The Public Health Agency of Sweden reported psychosomatic disorders, performance anxiety related to school, and differences in the socioeconomic background as factors contributing to increasing mental health problems in youth [4].

Previous studies of suicide rate trends and suicide methods in all child (< 18 years) suicides are scarce [6–9] and lacking from Sweden. Commonly, limited age groups of minors [2, 10] or ages including late adolescence (18–19 years) [1, 11–17] and young adults (22–24 years) [18] have been investigated, but suicides in younger children (< 15 years) were presented only sporadically in official national reports [5].

The present nationwide study of child suicides explores the suicide rate and demographic factors of age and sex in a 19-year-long period (2000–2018). Finally, the study aims to offer evidence and knowledge about child suicide methods and to inform child suicide prevention strategies.

## Materials and methods

Data were retrieved from the database of the National Board of Forensic Medicine regarding all registered suicides in children (under 18 years of age) in Sweden from 2000 through 2018. The database contains information from the police reports (circumstances) and the medicolegal autopsy (autopsy and toxicological findings, cause, and manner of death). In all, 416 child suicides were found and these data are included in the present study.

Variables collected were age, sex, date and place of death, presence of suicide note, information about survivability, and cause of death. Data was presented in two age groups (< 13 years and 13–17 years). The Swedish national guidelines for youth health and development define teenage starting at 13 years with age-specific developmental and health issues [19], hence why the cutoff age in the age groups was set at 13 years. In cases where firearm injury was the cause of death, information if the child was licensed/unlicensed firearm owner (yes/no), if the firearm involved was licensed (yes/no) and stored in the safety locker (yes/no), was received via the Swedish Police.

SPSS (version 26) for Windows was used in statistical data analyses. *χ*^2^ test and Fisher’s exact test were applied for the comparison of frequencies (method and sex, suicide seasons). The Poisson regression model was used for the analysis of the relationship between the number of child suicides and time (years), adjusted for child population size. The goodness-of-fit test did not show under- or overdispersion in the Poisson model. Data about child population size was retrieved from Statistics Sweden, the authority responsible for official statistics in Sweden. Statistical significance was defined as a *p*-value < 0.05.

## Results

### Mortality and demographics

**Fig. 1:**
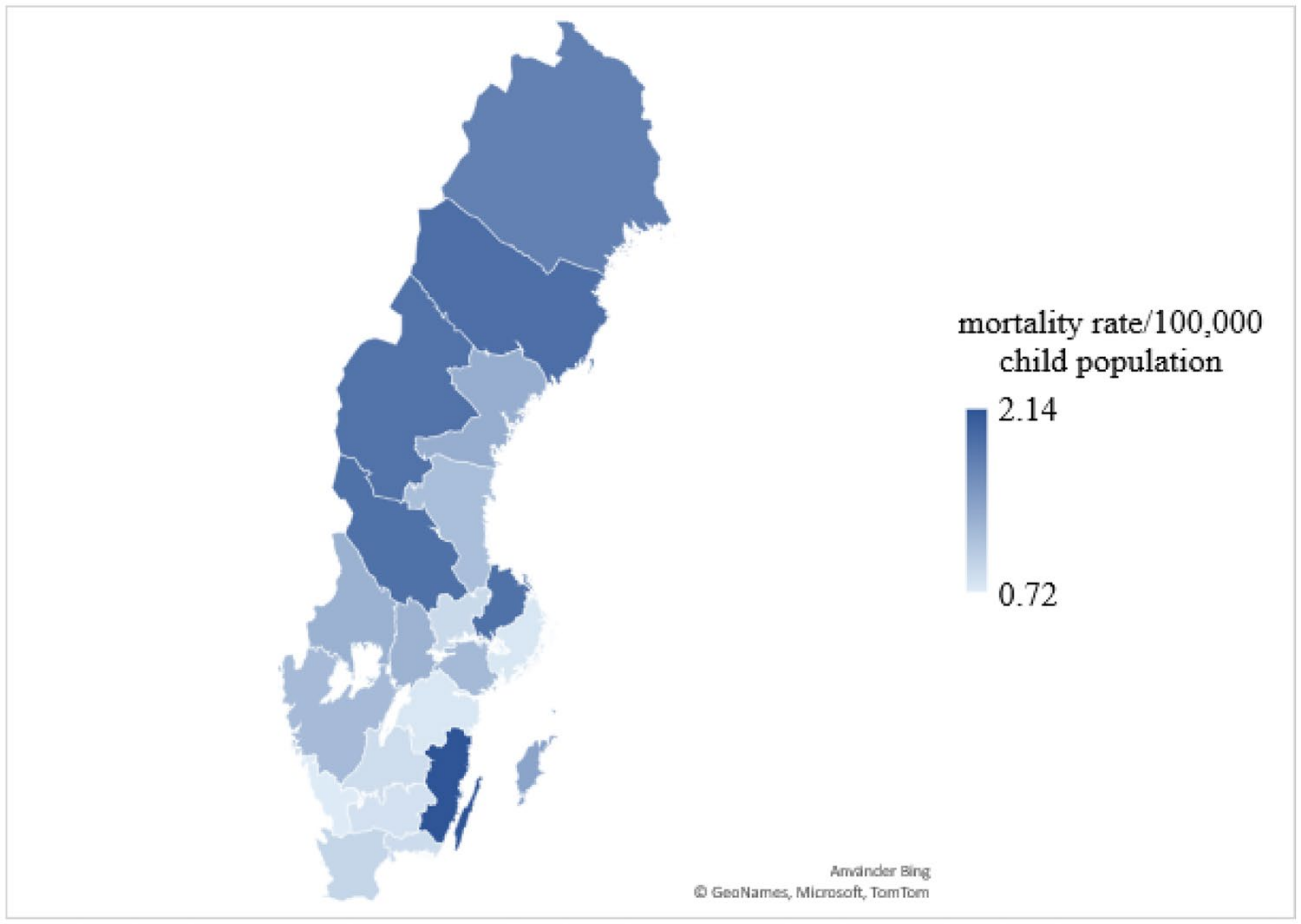
Child (< 18 years) suicide rate in 21 Swedish counties in 2000–2018, per 100,000 child population of each county

**Fig. 2:**
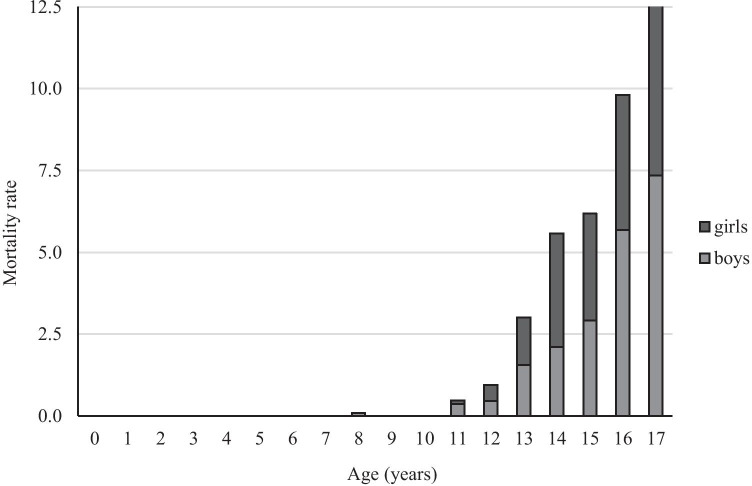
Child (< 18 years) suicide rate as the number of deaths per 100,000 children-years by sex and age, Sweden 2000–2018

**Fig. 3:**
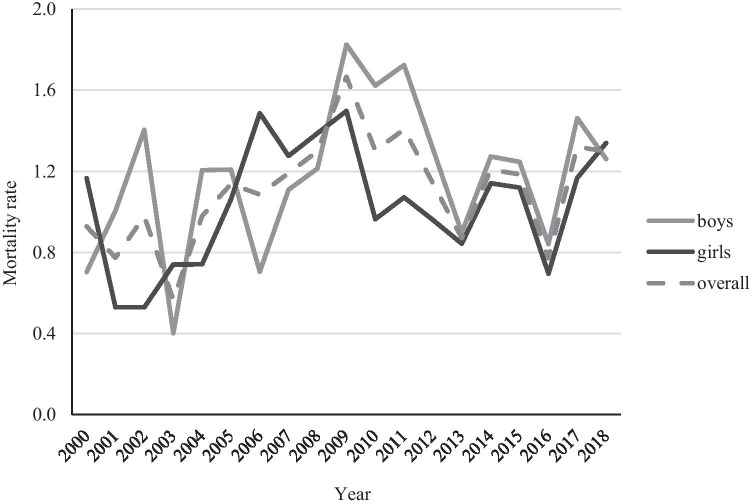
Child (< 18 years) suicide rate per 100,000 children-years by sex and overall, Sweden 2000–2018

In total, 416 child suicides were found in the period from 2000 through 2018. The suicide rate was, on average, 22 suicides/year or 1.1 per 100,000 child population/year (mean child population during the study period was 1.97 million). The highest rate of 2.14 child suicides per 100,000 child population was found in southeast Sweden, but also several northern counties had a high rate, > 1.5 per 100,000 child population (Fig. 1). The suicide rate increased with age (Fig. 2). The annual overall suicide rates and rates for boys and girls are illustrated in Fig. 3. The Poisson regression analysis (*df* = 1) showed that the time (years) was a significant and positive predictor of the number of child suicides (*B* = 0.021, *S.E.* = 0.012, *p* < 0.001), adjusted for child population size. The incidence rate ratio (*IRR* = 1.022) indicates that there was a 2.2% increase in the number of child suicides by each successive year.

The mean age was 16 years for both sexes (median 16 years, *SD* 1.5), 15 years for girls (median 16, *SD* 1.4), and 16 years for boys (median 16, *SD* 1.6). Boys accounted for 55% of all study cases. The majority of the children who died by suicide (96%) were teenagers (Table 1) and one-quarter (26%) were younger than 15 years.

**Table 1:** Suicide methods in minors < 18 years of age in Sweden 2000–2018, by age and sex

Suicide method	Girls *n* (% of all females)	Boys *n* (% of all males)	*p*-value	< 13 years *n* (% of all study cases)^3^	13–17 years *n* (% of all study cases)^3^	Total *n* (%)
Hanging	112 (59)	133 (59)	0.890^1^	11 (2.6)	234 (56.3)	245 (58.9)
Jumping/lying in front of a moving object	37 (20)	44 (20)	0.960^1^	2 (0.5)	79 (18.9)	81 (19.5)
Jump from a height	20 (11)	15 (7)	0.146^1^	2 (0.5)	33 (7.9)	35 (8.4)
Firearm	3 (2)	25 (11)	< 0.001^1^	1 (0.2)	27 (6.5)	28 (6.7)
Poisoning	11 (6)	6 (3)	0.103^1^	0 (0)	17 (4)	17 (4.1)
Drowning	4 (2)	2 (1)	0.417^2^	0 (0)	6 (1.4)	6 (1.4)
Suffocation	1 (0.5)	2 (1)	0.978^2^	0 (0)	3 (0.7)	3 (0.7)
Burns	1 (0.5)	0 (0)	0.999^2^	0 (0)	1 (0.2)	1 (0.2)
Total *n* (%)	189 (100)	227 (100)	-	16 (3.8)	400 (95.9)	416 (100)

^1^*χ*^2^ test

^2^Fisher’s exact test

^3^Low power for testing the hypothesis due to small number of cases < 13 years age

### Suicide methods and circumstances

The most common suicide methods were hanging (59%) and jumping or lying in front of a moving object (20%) (Table 1). Jumping from a height was the third (8%) and firearm suicide was the fourth most common suicide method (7%). Firearm suicide was uncommon among preteens (< 13 years) and girls. No cases of poisoning, drowning, or suffocation were found among preteens. A comparison between sexes indicated a statistically significant difference only for firearms (*p* < 0.001) (Table 1). Poisoning was more common among girls (65% of all poisonings), but this sex difference was not statistically significant (Table 1). Among poisonings, 88% were caused by licit drugs and 12% by carbon monoxide. One child died by burning.

The majority (94%) of the children was found dead, and the remaining minors died in hospital. In 58/416 (14%) cases, a suicide note was found. Twenty children (5%) were living in a residential care home when the suicide occurred.

Many suicides (31%) occurred in the autumn months (September–November), followed by 25% in the spring season (March–May). The summer (June–August) and winter (December-February) seasons each had equal shares of suicides (22%); both of these seasons had a significantly lower number of suicides than the autumn and spring months (*χ*^2^ = 5.538, *df* = 1, *p* = 0.019).

### Firearms

Firearms were involved in 28 of the suicides; 89% (25/28) of the children had a gunshot wound to the head and 11% (3/28) to the chest. The firearm was licensed in at least 86% (24/28) of the cases; in the remaining four cases, this aspect was unknown. In 96% (27/28), the minor was not a licensed firearm owner. Almost all firearms (96%, 27/28) were long-barreled, and the firearm was kept in a safety locker in 64% of the cases (18/28), not stored in a safety locker in 4% (1/28), and unknown in the remaining cases. Firearm suicides were spread over 15 of the 21 counties. The highest share of all firearm suicides was found on the island of Gotland (33%) and in Västmanland (22%).

## Discussion

This Swedish national study represents unique epidemiological research of suicide deaths in children during a 19-year-long period and offers evidence and aspects that should permeate specific prevention strategies for child suicides in a western setting.

### Mortality, demographics, and seasonal variation

Reported international variations of child and adolescent suicide rates and methods [1, 2] may be explained by differences in sociodemographic, health and cultural factors, prevention efforts, and availability of suicide means. In line with global statistics, the suicide rate is lower among Swedish children than among adults [5]. Overall, child mortality is decreasing in Sweden, mostly due to the decline in transport-related and other unintentional deaths [20]. Consequently, child injury mortality patterns have changed from unintentional injury predominance to more equal shares of intentional and unintentional injury in 1999–2012 [21]. Escalation of mental health issues in youths and an increased suicide rate among 15–24-year-olds in Sweden [5] makes an increasing number of child suicides to be expected.

A higher suicide rate in the northernmost counties may reflect a higher overall suicide rate in these counties [22] but, in spite of the highest child suicide rates, Uppsala and Västerbotten counties had the lowest overall suicide rates of all counties [22]. One-third of teenage deaths in the northern counties were intentional, predominantly suicides, with the highest rates among 16–19-year-old boys and with hanging, firearm injury, and intoxication as the most common methods [11]. Moreover, an interview study emphasized the cultural and sociopolitical aspects important for teenage suicides in northern Sweden, where the majority occurred in rural and depopulated geographical areas [23].

It has been suggested that the suicide rates can be underestimated due to the classification of the manner of death as undetermined or unintentional [2]. The classification issue may be linked to a suicide-related social stigma and/or misinterpretation of the child’s maturity and understanding of death [2]. Minors’ act of ending their own life may be seen rather as a deliberate means of self-harm than a suicidal act. The annual number of deaths with an undetermined manner of death was, however, much lower (2 deaths/year) among 0–14-year-olds than among 15–19-year-olds (8 deaths/year) in 2000–2018 [24]. Although the official mortality statistics present 15–19-year-olds as one age group, underestimation of child suicides might be limited.

An increasing suicide rate with age is an expected finding [7, 16, 25, 26] and can be partly explained by increased social pressure in the upper teenage years [12] and a higher prevalence of mental health issues among older children [25, 27]. Additionally, a low suicide rate in younger children and preteens was suggested to reflect age-related cognitive limitations in understanding the concept of death [28, 29] and in practical planning of the suicide act [12]. Children are said to understand death and its concept of irreversibility, as well as the concept of suicide [29] by the age of 8–9 years; before this age, they seem to consider death as reversible [28]. Accordingly, the younger child’s maturity and capability to act should not be underestimated [29, 30].

Although male preponderance to die by suicide is expected to grow with increasing age [22, 25, 26], the share of boys in the present study was only slightly higher than that of girls. In contrast, boys accounted for 75% of child suicides [31] and 85% of firearm suicides among 10–18-year-olds in the USA [32]. Firearm availability and the fact that males tend to use more lethal suicide methods could partly explain the appearance of an earlier sex asymmetry in the USA than in, e.g., Sweden.

The share of suicides was significantly lower during the summer and winter months—when Swedish children have longer school breaks—than in the autumn and spring months. Similarly, an Austrian study presented lower suicide rates during the summer months, suggesting a possible relationship with reduced school stress [16]. However, a causal relationship cannot be concluded, and further investigations of precipitative factors in the seasonal analysis are necessary.

### Suicide methods

Due to similarities in availability and lethality of the suicide means, some suicide method patterns have been described in clusters of countries [33]. Such a cluster analysis of suicide methods in 101 countries (2000–2009) and for 10–19-year-olds showed that Sweden was in the cluster with a mixture of methods, but with a predominance of hangings [33]. Hanging was a leading method also in England [34], Ireland [18], Canada [26], the USA [33], and Mexico [9]. Moreover, it was the most common method among the youngest, < 12 years of age, in the USA [10], as in the present study. Recently, an international review confirmed a high hanging prevalence ranging from 48–90% of child suicides [30].

A change in suicide method among children, from poisoning to hanging, has been explained by general trends with a decrease of CO poisoning in car exhaust fumes since cars became equipped with catalytic converters [35]. Suicides due to gas poisonings decreased in Sweden in the overall population from 1985 through 2004 and a shift from gas poisoning and drowning to hanging as a suicide method has been noticed, still with poisonings slightly more frequent than hangings [36]. Thus, increased use of hanging among children may reflect a general trend. Contrary to the small share of poisonings found in child suicides, poisoning as a “less violent” method is considerably more prevalent in adults, accounting for approximately one-third of all adult suicides [5, 36]. The small share of poisonings (4%) in the present study was in the lower interval (4–30%) of methods reported in previous studies [30, 34] that is possibly related to the change of methods and a restriction of drugs availability occurring before the study period.

Accordingly, the distribution of suicide methods among children found here does not imitate those in the general population [5]. Official national reports are masking child suicide data by age grouping together with adults, inhibiting an accurate comparison of methods between children and adults [22]. The preference to choose highly lethal methods in most of the suicides in the present study is a worrying fact. These methods together with firearms are more attributed to impulsiveness compared to other methods [37]. The impulsive attributes in suicide attempts and poor coping with stress seem also to be more common in children [30].

As reported in an older Swedish study, boys used violent methods (hanging, jumping, and firearms) more often than girls, but a change from non-violent to violent methods was observed for girls in the 1980s [17]. The share of violent methods among girls (91%) found here is higher than 36% found in the 1980s (among < 19-year-olds) [17]. Violent methods were used more often than non-violent methods by both sexes in the present study.

### Firearms

Internationally, firearm usage was the second most common method among children < 14 years old [30], but only the fourth most common method in the present material, particularly used by older children. In the USA, firearm suicides accounted for almost half of all suicides among 10–19 years old in 2017 [31] and safe firearm storage was suggested as a prevention measure [32]. Differences in firearm availability may explain such variations between countries. Swedish firearm legislation is very restrictive, limiting firearm licenses to persons > 18 years of age and requiring mandatory firearm storage in safety lockers [38]. Firearms can, only in exceptional circumstances, be licensed to individuals < 18 years, and a lower annual number of suicides due to firearms (1.5) among children than among Swedish adults (⁓ 100) [39] is thus expected.

Firearms are preferred by males as a suicide means both by adults [39] and children in the present study. In Sweden, licensed firearm owners are mostly male and are hunters [39, 40] and it is common that children follow their family in the hunt [41]. Research has shown that household availability and familiarity with firearms are important risk factors for suicides among youths in the USA [42, 43] and Australia [44]. Minors’ choice of the firearm as a method can be related to the availability of, and familiarity with, firearms also in a European setting.

In the USA, handguns accounted for two-thirds of firearms in child suicides and were mostly kept unlocked [32]. In contrast, the majority of firearms in the present study was long-barreled and stored in safety lockers, reflecting the dominance of rifles and shotguns for hunting and target shooting among Swedish gun owners [40], the strict licensing of handguns, and the strict storage regulation [38]. In spite of these storage restrictions, 28 children died from self-inflicted intentional firearm injuries in the study period. Most firearms were licensed, as in unintentional firearm deaths among Swedish children [45]. In conclusion, although stored in a locker, guns at home are associated with a higher risk of firearm suicide [42], and the locker’s keys or code must be kept unavailable.

### Strengths and limitations

A longitudinal design and analysis of accurate national data represent the strengths of the present study. Furthermore, additional sources from the police authority were used for information about firearms involved in child suicides. According to Swedish laws and regulations, all unnatural deaths should undergo a medicolegal autopsy at the National Board of Forensic Medicine. Missing cases among suicides are few (internal data). The data are thus representative of child suicides in Sweden during the study period. The register data offers a good consistency in examined variables. However, a limitation of the retrospective study design is the non-consistency of information regarding firearm storage.

### Prevention

Prevention of child suicides is a challenging task including multiple aspects. Besides universal prevention strategies such as restriction of means, efforts should reflect age-specific issues and should be adopted to specific local conditions. Restriction of suicide means at railways, bridges, and firearms must be targeted, representing common prevention means for both children and adults. Hanging as a dominant means of suicide is challenging to prevent due to its wide availability and increasing societal acceptance [14, 33]. Yet, prevention can include, e.g., improved primary prevention, mitigating the risk factors for suicide, and interventions affecting broader general conditions and risk groups among children.

Precipitating factors differ between children and adults and may be related to school problems such as bullying [46–48], underachievement, disturbances in relation with parents, and/or family violence [30]. In the present study, 5% of the children who died by suicide had been placed in a residential foster home. Children who are adopted [49–51] or living in a care home [52] are also at a higher suicide risk than others. As for adults [53], previous suicide attempts, psychiatric disorders, and traumatic events are some of the risk factors [30, 54]. High-quality psychiatric health care for children, prevention programs at school, and strengthening of family relations [55] are evidently important prevention strategies.

## Conclusions

Child suicide prevention represents an important national priority, and the mortality trend needs to be inverted. Further research is necessary to enlighten apparent regional differences in child suicide rates. Official mortality statistics should be improved for surveillance of suicide trends regarding minors separately from suicide trends for older ages. Risk and vulnerability factors and prevalence of suicide methods differ from those in adults; hence, protective strategies need to be tailored explicitly for the child population and for the choice towards more lethal means.

## Data Availability

Data and material are available for researchers who meet the criteria for access to confidential data.

## Abbreviations

CI: Confidence interval
CO: Carbon monoxide
SD: Standard deviation
SPSS: Statistical Package for the Social Sciences
USA: United States of America
WHO: World Health Organization
